# Veno-Arterial Extracorporeal Membrane Oxygenation for Septic Cardiomyopathy due to* Legionella *Pneumonia after Influenza Virus Infection

**DOI:** 10.1155/2018/6973197

**Published:** 2018-10-22

**Authors:** Motohiro Asaki, Takamitsu Masuda, Yasuo Miki

**Affiliations:** Department of Emergency Medicine, Emergency and Critical Care Center, Fujieda Municipal General Hospital, Shizuoka, Japan

## Abstract

A 57-year-old man presented to the emergency department with fever and progressive altered level of consciousness of 5 days' duration. Three days before admission, influenza A was diagnosed at a clinic. On admission, his vital signs were unstable. Pneumonia was diagnosed through chest computed tomography, and urinary* Legionella* antigen test was positive. A diagnosis of septic shock due to* Legionella* and influenza pneumonia was made, and critical care management was initiated, including mechanical ventilation and vasopressors. However, tachycardia did not improve, left ventricular ejection fraction was 20%, and circulatory insufficiency progressed. Therefore, considering the involvement of septic cardiomyopathy and cardiogenic shock, veno-arterial extracorporeal membrane oxygenation (VA-ECMO) was initiated for circulation assistance on day 3 since admission. Tachycardia and myocardial dysfunction improved by day 8, and VA-ECMO was withdrawn. Subsequently, nutrition management and rehabilitation were performed, and the patient was transferred to a recovery hospital on day 108. VA-ECMO may be beneficial when concomitant with circulatory assistance in uncontrollable cases of septic cardiomyopathy using catecholamines and *β*-blockers. It may be necessary to adopt VA-ECMO at an appropriate time before the patient progresses to cardiopulmonary arrest.

## 1. Introduction

*Legionella* pneumonia occasionally leads to severe septic shock and multiple organ dysfunction and is known to follow a lethal course. In 2008, Von Baum et al. reported that the mortality rate of* Legionella* pneumonia was 14% [1]. Furthermore, reversible myocardial depression in patients with septic shock was first described in 1984 by Parker et al., as septic cardiomyopathy [2]. Veno-arterial extracorporeal membrane oxygenation (VA-ECMO) is an extracorporeal circulation device used for severe cardiogenic shock, and Brechot et al. reported the effectiveness of VA-ECMO on septic shock with cardiovascular dysfunction [3]. This case report describes the successful treatment of severe* Legionella* pneumonia that led to septic shock and severe septic cardiomyopathy by timely and proper introduction of VA-ECMO.

## 2. Case Presentation

**Figure 1:**
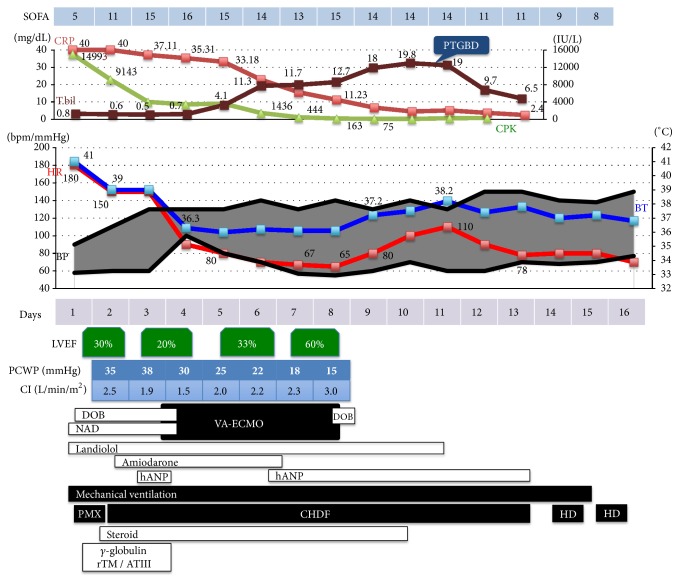
Patient's clinical course in the intensive care unit.

A previously healthy 57-year-old man presented to the emergency department with fever and progressive altered mental status for 5 days. Three days before admission, he was diagnosed with influenza A at a clinic, for which oseltamivir was prescribed. Upon arrival at the emergency department, his vital signs were unstable: respiratory rate, 40 breaths/min; heart rate, 153 beats/min (irregular); blood pressure, 96/70 mmHg; body temperature, 39.9°C; and Glasgow Coma Scale (GCS), 11/15 (E3V3M5). On physical examination, an oral mucosa was dry and coarse crackles in the left lung were documented, but there were no heart murmur and no lower edema. Blood examination results were as follows: white blood cell count (10100/*μ*L); hemoglobin (15.7 d/L); platelet count (12.8 × 10^3^ /*μ*L); C-reactive protein (36.82 mg/dL); creatine kinase (3181 IU/L); procalcitonin (19.58 ng/mL); and brain natriuretic peptide (123 pg/mL). Urinary* Legionella* antigen test was positive, while rapid influenza A and B antigen tests were both negative. Arterial blood gas analysis yielded the following findings: pH 7.54, pCO_2_ 25.8 mmHg, pO_2_ 81.2 mmHg (O_2_ 10 L/min reserver mask), HCO_3-_ 21.1 mmol/L, and lactate 2.0 mmol/L. A consolidation was observed in the left lung field by chest X-ray and chest computed tomography (CT). Electrocardiogram initially documented atrial fibrillation (AF), while echocardiogram revealed left ventricular ejection fraction (LVEF) of approximately 30%. Due to the presence of* Legionella*, pneumonia, and qSOFA of 3 points, it was diagnosed with* Legionella* pneumonia and septic shock. He was transferred to the intensive care unit (ICU), intubated, and started with mechanical ventilation management and intensive care. His progress in the ICU is shown in Figure 1.

Tazobactam/piperacillin and levofloxacin for* Legionella* pneumonia and peramivir for suspected influenza pneumonia were initiated intravenously. For septic shock, noradrenaline at 0.06 *μ*g/kg/min, hydrocortisone at 200 mg/day, and intravenous immunoglobulin were started. In view of AF, landiolol was started at 1 *μ*g/kg/min and then increased to 10 *μ*g/kg/min. Moreover, due to the low ventricular contractility, additional dobutamine at 3 *μ*g/kg/min was initiated. The patient was then given polymyxin B-immobilized fiber and continuous hemodiafiltration (CHDF) for acute kidney injury (AKI) and suspected endotoxin shock (endotoxin level was later observed to be high, at 139.7 pg/mL). On day 3 of hospitalization, we performed cardioversion several times for AF and there was a temporary return to normal sinus rhythm (SR); however, it immediately returned to AF. Transthoracic echocardiography showed that LVEF worsened to 15%, and the left ventricular end-diastolic/systolic diameter (LVDd/Ds) was increased to 61/55 mm. On the night of day 3, metabolic and respiratory acidosis progressed due to circulatory failure (pH 7.138, pO_2_ 70.4 mmHg, pCO_2_ 68.4 mmHg, BE -8.7 mmol/L, lactate 2.4 mmol/L) (ventilator mode: pressure control ventilation: FiO2 70%, RR 20/min, PEEP 9 cmH_2_O, PC 15 cmH_2_O). Thus, the patient was diagnosed with cardiogenic shock due to septic cardiomyopathy. This required introduction of VA-ECMO and catecholamines were discontinued. Subsequently, the acidemia ameliorated and hemodynamic circulation stabilized. AF reverted to normal SR on day 5. And on day 7, LVEF recovered to 60%. Therefore, it stabilized hemodynamics by infusion, a little catecholamine, and VA-ECMO was discontinued on day 8.

While VA-ECMO was in progress, the patient developed progressive jaundice; this appeared to be a complication of VA-ECMO because of mechanical hemolysis (on day 7, total/direct bilirubin: 13.3/9.6 mg/dL, LDH 3077 IU/L, Hb: 8.1 g/dL). Therefore, after VA-ECMO withdrawal, we expected that total bilirubin will be decreased, but no improvement was observed (max total/direct bilirubin: 19.8/15.8 mg/dL). Cholecystitis was detected via an abdominal echography. Following percutaneous transhepatic gallbladder drainage performed on day 11, the bilirubin level declined. Subsequently, on day 15, the patient's general condition stabilized, and he was extubated. However, CHDF was switched to hemodialysis (HD) as the patient's anuric state due to AKI persisted. After 16 days, the patient was discharged from the ICU.

An arteriovenous fistula was created as a continuation of maintenance HD was necessary. Although, in the ICU, the patient had been on initiated enteral nutrition (EN), he was unable to tolerate EN due to intestinal dysfunction advancement. Therefore, he was initiated on total parenteral nutrition. Long-term rehabilitation intervention was also required due to ICU-acquired weakness (ICU-AW), because of long-term sedation and muscle relaxation during extracorporeal support. After 70 days, dialysis was discontinued; the patient was able to eat independently at approximately 90 days. He was transferred to a recovery hospital on day 108 and underwent complete social reintroduction at 5 months after discharge from our hospital.

## 3. Discussion

In the present case report, we treated a 57-year-old man with severe* Legionella *pneumonia using VA-ECMO. Interestingly, through this case we could properly judge VA-ECMO adaptation by evaluating appropriate shock.

Septic cardiomyopathy (or sepsis-induced cardiomyopathy) has been recognized in sepsis treatment since first reported by Parker in 1984 [1]. In a study by Sato et al. [4], the following characteristics were stated as clinical features: left ventricular dilatation, depressed left ventricular ejection fraction, and recovery within 7-10 days. The causes of septic cardiomyopathy are chemical mediators, specifically endotoxin, cytokines, and nitric oxides, as well as induced mitochondrial dysfunction and downregulation of *β*-adrenergic receptors. In the present case, although LVEF declined from the time of the hospitalization, left ventricular expansion was not initially considered; treatment was initiated based on the opinion that vasoplegic shock and intravascular depletion were the main cause. However, on day 4, LVDd/Ds showed clear left ventricular expansion at 61/55 mm, and the patient was diagnosed with severe septic cardiomyopathy.

We needed to determine the possibility of acute myocarditis. There was no elevation in myocardial enzyme levels (troponin I 0.228 ng/ml on day 3 and CK-MB 12 IU/L on day 4), and myocardial edema and pericardial effusion were not observed on echocardiogram even when left ventricular dysfunction occurred. Therefore, acute myocarditis was judged to be negative.

During treatment, Gattinoni et al. reported that using dobutamine and dopamine as support for the cardiac index failed to reduce morbidity and mortality among critically ill patients [5]. Morelli et al. suggested that *β*-blockade could be associated with a reduction in the heart rate without adverse effects and that this could help improve survival [6]. The effectiveness of *β*-blokers for sepsis is still under discussion, but in this case the use of landiolol for heart rate control from the time of hospitalization may have been a little effective. However, dobutamine administered for cardiac dysfunction with uncontrollable tachycardia may have been an exacerbating factor.

There are several case reports on the successful use of VA-ECMO as rescue therapy for unresponsive patients with severe cardiogenic shock with septic cardiomyopathy. Septic shock is typically warm shock due to peripheral vasodilation and peripheral vascular hyperpermeability. However, among shocks associated with sepsis, diagnosis of the involvement of cardiogenic shock (= septic cardiomyopathy) in addition to septic shock is key to judging the introduction of VA-ECMO. Sato et al. reported that mechanical support with ECMO might be a therapeutic option; however, further studies are required to confirm whether it is truly effective in septic cardiomyopathy [4].

The timing of VA-ECMO introduction is also controversial. ECMO is considered an invasive and excessive introduction that may worsen prognosis due to complications. In this case, we decided to introduce VA-ECMO because it was judged that there was a high possibility of falling into CPA by prolongation of shock, based on the progression of both respiratory and metabolic acidosis. Securing an artery/vein for VA-ECMO is necessary; however, doing this following cardiopulmonary arrest (CPA) will lead to a puncture without pulsation, thus increasing the frequency of punctures and complications associated with it. Therefore, it is important to secure an artery/vein sheath prior to the introduction of VA-ECMO and introduce it in the state of shock before CPA.

In conclusion, we treated a patient with severe* Legionella* pneumonia using VA-ECMO. We wish to highlight the importance of timely and appropriate introduction of VA-ECMO, with the recognition of the involvement of septic cardiomyopathy by evaluating cardiac function in septic shock.

## Abbreviations

AT III: Antithrombin III
BP: Blood pressure
BT: Body temperature
CHDF: Continuous hemodiafiltration
CPK: Creatine kinase
CRP: C-reactive protein
DOB: Dobutamine
hANP: Human atrial natriuretic peptide
HD: Hemodialysis
HR: Heart rate
LVEF: Left ventricular ejection fraction
NAD: Noradrenalin
PMX: Polymyxin-B-immobilized fiber
PTGBD: Percutaneous transhepatic gallbladder drainage
rTM: Recombinant human soluble thrombomodulin
SOFA: Sepsis-related organ failure assessment
T. bil: Total bilirubin
VA-ECMO: Veno-arterial extracorporeal membrane oxygenation
PCWP: Pulmonary capillary wedge pressure
CI: Cardiac index.
